# Colleague appraisal of Australian general practitioners in training: an analysis of multisource feedback data

**DOI:** 10.1186/s12909-022-03559-5

**Published:** 2022-06-24

**Authors:** Caitlin Vayro, Ajit Narayanan, Michael Greco, Neil Spike, Jan Hanson, Ben Mitchell, Dale Hanson, Rebecca Stewart

**Affiliations:** 1 General Practice Training Queensland, Brisbane, Australia; 2 School of Public Health, Faculty of Medicine, The University of Queensland, Brisbane, QLD Australia; 3 School of Engineering, Computer and Mathematical Sciences, Auckland University of Technology, Auckland, New Zealand; 4 School of Medicine, Griffith University, Brisbane, QLD Australia; 5 CFEP Surveys, Everton Park, QLD Australia; 6 Eastern Victoria General Practice Training, Hawthorn, VIC Australia; 7 Department of General Practice and Primary Health Care, The University of Melbourne, Carlton, VIC Australia; 8 School of Rural Health, Monash University, Victoria, Australia; 9 Northern Territory General Practice Education, Darwin, NT Australia; 10 Primary Care Clinical Unit, Faculty of Medicine, The University of Queensland, Brisbane, Australia; 11 College of Public Health, Medicine and Veterinary Sciences, James Cook University, Townsville, QLD Australia; 12 The Royal Australian College of General Practitioners, Melbourne, Australia

**Keywords:** Professional development, Professionalism, Multisource feedback, GPs in Training, GP Registrars, Communication skills

## Abstract

**Background:**

Multisource feedback is an evidence-based and validated tool used to provide clinicians, including those in training, feedback on their professional and interpersonal skills. Multisource feedback is mandatory for participants in the Royal Australian College of General Practitioners Practice Experience Program and for some Australian General Practice Training Registrars. Given the recency of the Practice Experience Program, there are currently no benchmarks available for comparison within the program and to other comparable cohorts including doctors in the Australian General Practice Training program. The aim of this study is to evaluate and compare colleague feedback within and across General Practice trainee cohorts.

**Methods:**

Colleague feedback, from multisource feedback of Practice Experience Program participants and Australian General Practice Training Registrars, collected between January 2018 and April 2020, was compared to identify similarities and differences. Analyses entailed descriptive statistics, between and within groups rater consistency and agreement measures, principal component analysis, t-tests, analysis of variance, and psychometric network analysis.

**Results:**

Colleague ratings of Practice Experience Program participants (overall average 88.58%) were lower than for Registrars (89.08%), although this difference was not significant. ‘Communication with patients’ was rated significantly lower for Practice Experience Program participants (2.13%) while this group was rated significantly better for their ‘Ability to say no’ (1.78%). Psychometric network analyses showed stronger linkages between items making up the behavioural component (compared to the items of the performance and self-management components, as found by principal component analysis) for Practice Experience Program participants as compared to Registrars. Practice Experience Program participants were stronger in clinical knowledge and skills as well as confidentiality, while Registrars were stronger in communicating with patients, managing their own stress, and in their management and leadership skills.

**Conclusions:**

The multisource feedback scores of doctors undertaking the Practice Experience Program suggests that, while all mean values are ‘very good’ to ‘excellent’, there are areas for improvement. The linkages between skills suggests that Practice Experience Program doctors’ skills are somewhat isolated and have yet to fully synthesise. We now have a better understanding of how different groups of General Practitioners in training compare with respect to professional and interpersonal skills. Based on the demonstrated differences, the Practice Experience Program might benefit from the addition of educational activities to target the less developed skills.

## Background

Multisource feedback (MSF) is a valued educational feedback and formative assessment tool used to facilitate reflection on communication skills, teamwork, and professionalism [1–3]. MSF comprises patient and colleague feedback and self-appraisal, using reliable and validated measures [1, 2]. The Royal Australian College of General Practitioners (RACGP) pathways to General Practitioner (GP) Fellowship (i.e., satisfactorily completing the education/training pathway to become a vocationally registered specialist in General Practice) include two different programs. The programs are the Practice Experience Program (PEP) where MSF is mandated, and the Australian General Practice Training Program (AGPT) where MSF is variably implemented by the ten Training Organisations delivering GP training on behalf of the RACGP [4–6]. Currently, the performance of AGPT Registrars is compared to established MSF benchmarks (i.e., the descriptive statistics that indicate the performance of the cohort: mean, minimum, maximum, and standard deviation) in the AGPT cohort. More recently, the PEP has been introduced for doctors who are ineligible to apply, do not obtain a place, or choose not to apply to the AGPT program [5, 7]. The PEP is a self-directed education program rather than a structured training program (see below), with a cohort predominantly consisting of doctors who have gained their primary medical degree outside of Australia (90.9%) and are already working in General Practice [8]. While the MSF is a required component of the PEP, as yet PEP General Practitioners in Training (GPiT; hereon encompassing PEP participants and AGPT Registrars) do not have benchmarks to understand the comparative performance of individuals within this group, or between GPiT completing different programs. Feedback scores can be useful to understand the performance of a single doctor. However, greater utility arguably occurs through comparison with peers. Therefore, this research aimed to ascertain benchmarks for PEP GPiT and compare their performance with AGPT GPiT, specifically focusing on the colleague feedback portion of MSF.

MSF comparisons are important to enable an understanding of an individual’s performance with respect to their peers, as well as how different cohorts of doctors compare. For example, Narayanan et al. [2] have demonstrated that doctors who undertook MSF as remediation required by the Australian Health Practitioner Regulation Agency (AHPRA) received lower ratings for their professionalism from colleagues than other groups of GPs, including AGPT GPiT. There is also evidence that colleague feedback scores can be predictive of the future need for remediation and summative assessment performance for GPiT [9, 10]. Thus, understanding the score profile of GPiT on programs to RACGP Fellowship allows for the identification of concerns about a doctor’s performance and provision of appropriately tailored support.

Understanding feedback scores is particularly important for medical educators delivering the PEP, where the participants are engaged in a relatively new, largely self-directed education program. In comparison, the AGPT program is well-established, and includes training, prescribed supervision, monitoring of progression and mandated remediation (where indicated) in a more formal and structured approach than the PEP. Despite program differences, benchmarking of doctors training towards GP Fellowship would benefit from documenting across both programs, given that the competencies required at the point of Fellowship in the communication and professionalism domains of practice are the same. It should not be assumed that the benchmarks for each GPiT group are equivalent given that the performance of PEP GPiT has not yet been examined or compared with those on the AGPT program.

There are other factors that might influence the performance of PEP GPiT that warrant consideration. The criteria for the AGPT program, particularly the need to be an Australian citizen or permanent resident, or a New Zealand citizen, likely mean that the PEP is more commonly undertaken by overseas trained doctors (OTDs; as is shown by the General Practice: Health of the Nation 2021 report [8]), who are needed to help address the workforce shortage of GPs in rural and regional Australia [11]. Indeed, the purpose of the PEP assumes that many of the participating doctors have gained their primary medical qualifications outside Australia [5]. Therefore the performance of the PEP cohort cannot be assumed to be comparable to AGPT GPiTs, as there are reportedly differences in demographic profiles and cultural and communication approaches [12]. For example, Laurence et al. [13] found that OTDs in Australia were older with a greater amount of time since obtaining their medical degree. There were also several personality differences found between Australian and OTDs, where OTDs expressed lower novelty-seeking, persistence, self-directedness, cooperativeness, and total resilience, which are suggested to reflect their culture and prior medical training. As such, these differences need to be considered, given that it could impact a doctor’s perceived communication skills and professionalism. This again supports the need to understand MSF performance specific to this group.

Demographic factors seemingly impact progression through GP Fellowship programs as well as performance as a GP. There is evidence that completing medical training outside of Australia, being male, and aged 35 or over, are predictive of both a need for remediation and lower pass rate for GP Fellowship summative assessments [9, 10]. In addition, some of these factors have been associated with reduced quality of patient care [14]. Several reasons have been put forward to explain the demographic differences between domestic and international medical graduates such as difficulty with the English language, the process of migration and adjustment, differences in medical education, length of time since medical school graduation, the status and role of the physician, cultural approaches and beliefs, and family and financial obligations [12, 15]. These factors are likely to impact on both clinical knowledge and professionalism. Thus, it is important to understand the performance of PEP GPiT, who seemingly share some of these risk factors, to be able to provide additional assistance and tailored education, if necessary. As such, understanding PEP GPiT’s performance through colleague feedback and comparison to AGPT GPiT’s performance can facilitate interventions to enhance these important GP skills.

Understanding GPiT’s performance as rated by colleagues and in turn communication skills and professionalism is crucial, given the risks, implications, and opportunity for interventions to enhance future independent practice. The aim of this research is to examine the MSF performance of doctors undertaking programs to prepare for RACGP Fellowship as rated by their colleagues. That is, colleague ratings of the professionalism of each cohort of GPiTs will be aggregated to assess benchmarks and compared. Further, this examination will also depict the psychometric network of each GPiT cohort’s MSF performance, to show how the associated skills cluster for each cohort. By extension, we also aimed to draw inferences regarding beneficial additional supports or interventions for GPiT. The results of patient feedback and self-appraisal will be reported in additional publications.

## Methods

### Participants

The sample comprised two groups of doctors undertaking programs towards Fellowship with the RACGP. The surveys are completed by their colleagues (doctors, other healthcare professionals, and managerial/administrative staff). In total, two sets of fully anonymised data were obtained as follows:

1. Group 1 consists of 265 doctors undertaking the PEP to RACGP Fellowship. For these 265 PEP GPiT, 3441 colleague responses were obtained
2. Group 2 consists of 97 doctors undertaking the AGPT program to RACGP Fellowship. For these 97 AGPT GPiT, 1289 colleague responses were obtained.

### Data collection

The University of Queensland Human Research Ethics Committee approved this study. The data were collected in the period between 1^st^ January 2018 and 30^th^ June 2020. The participants were undertaking the MSF process as part of their GP program requirements. The participants gave consent for their non-identifiable data to be used in research as part of the consent process to undertake the MSF process. The data custodian (CFEP Surveys, a professional health survey organisation) provided access to the de-identified data.

Participating GPiT were advised to nominate at least 15 colleagues with whom they work, including doctors, other healthcare professionals and managerial/administrative staff [1]. Nominated colleagues were then sent the questionnaire (Colleague Feedback Evaluation Tool [1, 2]) for completion, with a follow-up reminder, if required. The colleague questionnaire asks colleagues to rate their interactions with the target doctor on aspects of clinical competence, management, communication and leadership [1] There is a final question relating to overall ability. Table 2 contains a brief description of questionnaire items. All 19 items use a five-point Likert scale with labels ‘poor’, ‘fair’, ‘good’, ‘very good’, and ‘excellent’. Colleague anonymity was guaranteed for all responses provided.

The colleague questionnaire was completed online or as a paper postal survey. The online questionnaires were completed via a secure online web portal. The questionnaires were processed by CFEP Surveys. Online survey data validation and verification were conducted before being downloaded to in-house software systems; the same procedures were then carried out for the paper questionnaires after manual data entry. The dataset was exported as a Microsoft Excel Spreadsheet to an SPSS database (SPSS for Windows Version 25) and cleaned and checked prior to data analysis.

### Analysis

The measures use Likert scales, and to aid interpretation it has been assumed that the intervals between each scale point are equal and equate to percentages. This means that a ‘poor’ rating is equivalent to 20%, ‘fair’ to 40%, ‘good’ to 60%, ‘very good’ to 80%, and ‘excellent’ to 100%. This allows for parametric techniques that utilise descriptive statistics such as means, standard deviations, and variances to be calculated, and aligns with the presentation of previous MSF results [6]. To understand the doctors’ performance (i.e., the benchmarks), both raw and aggregate data is reported. That is, we examine both individual items, as well as mean scores for the colleague evaluation.

### Reliability and validity

The sampling method used impacts the internal consistency and reliability of the measure (often decided using Cronbach’s α). Such sampling means the data is unbalanced because the number of raters per ratee is variable, fully nested because raters have unique ratees, and uncrossed because raters only provide a single rating. Cronbach’s α is reported below as a measure of questionnaire reliability, but the results should be interpreted cautiously because the assumptions of its use are not met in this study (e.g., all raters are rating the same subject, object, or event). Therefore, this is complemented by a signal-to-noise ratio (SNR) formula for checking the reliability of the questionnaire data [16]. This formula combines raw and aggregated item, rater, and subject variances, with consideration to the average number of raters per ratee, to address the issues introduced by the sampling (i.e., that it is unbalanced, uncrossed, and fully nested data). Additionally, single measures intraclass coefficients (ICCs) are used to check for inter-rater reliability for this specific study. A two-way mixed effects model was chosen since each doctor is rated by a different set of colleagues who were specifically selected by the doctor from a larger population of possible colleagues and not drawn randomly. ICCs of 0.4–0.6, 0.6–0.8, 0.8 + are considered to show moderate, good and very good agreement, respectively [17].

Principal component analysis (PCA) is used here to determine if the criterion and construct validity of the colleague questionnaires are within accepted conventions. PCA is a data reduction technique for explaining variance in data using a smaller set of variables than the original variables or items. Varimax method is used for rotating and extracting the components, whereby each component has a small number of large loadings. The Kaiser–Meyer–Olkin (KMO) test is a sampling measure for indicating suitability for PCA. KMO values between 0.8 and 1.0 indicate that there are enough samples and sufficiently low variance for efficient identification of components, which is the case for the colleague feedback data (KMO = 0.97)[18] Bartlett’s test for sphericity is another measure for testing the suitability of data reduction which check for correlations between variables. A significant Bartlett test, as was found (p ≤ 0.001), indicates the variables are sufficiently correlated for PCA[18]. Three components were found accounting for 81% of the variance (Table 1), corresponding to performance (component 1), behaviour (component 2) and self-management (component 3).

**Table 1:** Principal component analysis reveals three components (clinical performance, behaviour, self-management) in colleagues’ ratings of PEP and AGPT GPiT

Items	Component		
	**1**	**2**	**3**
Clinical knowledge	0.77		
Clinical ability	0.79		
Communication with patients	0.81		
Compassion/empathy	0.77		
Colleague communication	0.66		
Teaching and training colleagues	0.70		
Punctuality and reliability		0.69	
Respect for colleagues		0.75	
Ability to say "no"			0.84
Awareness of limitations	0.63		
Team orientation	0.64		
Use of resources	0.68		
Ability to manage stress			0.55
Confidentiality		0.67	
Appearance and behaviour		0.78	
Respect to their own health		0.70	
Trustworthiness/honesty/probity		0.69	
Management/leadership skills	0.64		
Overall ability	0.77		
*Variance explained*	*36.65%*	*28.68%*	*15.49%*

Note: Only the highest component loadings shown for the 19 items

### Data analysis

Two analytical methods were used to compare the GPiT’s performance, analysis of variance (ANOVA) and t-tests. ANOVA is used to test for differences in item ratings and means, in and between, PEP and AGPT data. Independent samples T-tests are used in this study to examine whether item means differ between the two doctor groups. In addition, regression was used to control for the effects of demographic factors on colleague raw scores.

Psychometric network analysis [19] is a rapidly growing area used to statistically analyze and visually present patterns of mutual influence relationships between psychological and psychometric variables. Such relationships are depicted using network models and topologies (nodes and links) taken from mathematics and physics, with nodes representing variables or items, and links representing associations or pairwise interactions between variables and items. Such networks provide a model of how different variables reinforce each other. Network analysis is performed at the aggregated level in this study, with pairwise correlations between items used as the method of association and thicker lines indicating stronger relationships. Such networks provide mechanisms for understanding doctor performance at a systems level using all items and distinguish doctor groups through different item-interaction patterns. Summing the absolute inter-item correlations for each item results in a node ‘strength’ measure that can be useful for assessing the stability of such networks.

## Results

There were 3441 separate colleague responses (38% doctor, 60.8% other, 1.2% not declared; 65.5% female, 33.3% male, 1.2% not declared) to 265 PEP GPiT (mean number of colleagues per doctor = 12.98, SD 1.31, minimum 12, maximum 23,). There were 1289 separate colleague responses (41.4% doctor, 57.9% other, 0.7% not declared; 73.1% female, 26.1% male, 0.8% not declared) to 97 AGPT GPiT (mean number of colleagues per doctor = 13.57, SD 2.87, minimum 12, maximum 34,).

Questionnaire reliability using Cronbach’s alpha was 0.97 for PEP and 0.96 for AGPT, with an average inter-item correlation of 0.61 and 0.58, respectively, indicating high internal consistency of the questionnaire items and good consistency for measuring the same general underlying construct. The ICC for PEP was 0.60 and for AGPT was 0.57, indicating moderate to good agreement among the colleagues for interpreting the questionnaire items. Data reliability calculated using SNR was 0.82 for PEP colleagues, indicating that 82% of the data is likely to be true data with the rest due to noise and error from interactions between raters, items, and ratees. SNR data reliability was higher for AGPT colleagues at 0.90, indicating that 90% of the data was likely to be true data.

**Fig. 1:**
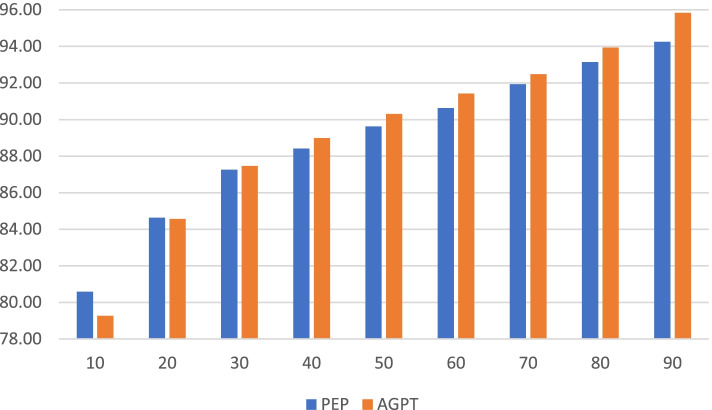
Comparison of PEP (*n* = 265) and AGPT (*n* = 95) doctors’ average score received from colleagues (y-axis) by percentile (x-axis). Note: The bottom percentile values are 80.58% and 79.26% for PEP GPiT and AGPT, respectively. The y-axis has been constrained to help make the differences clearer

When colleague scores were aggregated by doctor, the average score received by all doctors was in the ‘Very good’ to ‘Excellent’ range at 88.83% (Table 2), with AGPT GPiT scoring higher (89.08%) than PEP (88.58%). Although the difference in average score was not significant, scores on two specific items were significantly different. PEP GPiT received on average 2.13% lower scores for ‘Communication with patients’ and 1.78% higher scores for ‘Ability to say ‘no’’ (p ≤ 0.05). The highest scoring item was ‘Appearance and behaviour’ (93.59%) for PEP and ‘Trustworthiness/honesty’ for AGPT (94.24%). The lowest scoring item for both groups of doctors was ‘Ability to say no’ (82.99% for PEP, 81.20% for AGPT). PEP GPiT received significantly lower minimum scores than AGPT (62.2% vs 67.59%, p ≤ 0.01). There was a tendency for PEP GPiT to have an average score lower than AGPT for all percentiles except the 10^th^ and 20^th^ (Fig. 1).

**Table 2:** Colleague scores for PEP GPiT and AGPT GPiT for all 19 questionnaire items

	PEP GPiT (*n* = 265)				AGPT GPiT (*n* = 97)				
Item	Min	Mean	Std. error	Std. dev	Min	Mean	Std. error	Std. dev	*AGPT-PEP GPiT*
Clinical knowledge	56.00	88.17	0.41	6.71	67.14	89.10	0.68	6.66	*0.92*
Clinical ability	61.67	87.94	0.43	6.97	67.14	89.14	0.68	6.66	*1.20*
Communication with patients	58.33	87.55	0.52	8.45	71.11	89.68	0.80	7.83	***2.13***
Compassion/empathy	63.64	90.53	0.43	7.01	66.67	90.40	0.83	8.09	*-0.13*
Colleague communication	60.00	88.83	0.44	7.10	67.69	89.58	0.82	7.96	*0.75*
Teaching and training colleagues	52.50	83.31	0.47	7.58	62.00	84.34	0.77	7.53	*1.03*
Punctuality and reliability	60.00	88.89	0.47	7.62	47.27	90.17	0.88	8.61	*1.27*
Respect for colleagues	61.67	92.76	0.37	6.03	74.29	92.80	0.69	6.70	*0.04*
Ability to say "no"	62.22	82.99	0.38	6.11	68.57	81.20	0.67	6.57	***-1.78***
Awareness of limitations	61.67	87.22	0.41	6.60	68.33	87.66	0.73	7.13	*0.45*
Team orientation	60.00	87.41	0.40	6.55	66.15	88.14	0.73	7.14	*0.73*
Use of resources	61.67	87.76	0.39	6.27	73.33	89.02	0.64	6.27	*1.26*
Ability to manage stress	51.67	86.04	0.43	7.05	56.00	84.52	0.80	7.79	*-1.52*
Confidentiality	76.36	93.51	0.29	4.78	76.36	93.84	0.49	4.77	*0.33*
Appearance and behaviour	70.00	93.59	0.31	5.05	75.00	93.94	0.58	5.63	*0.36*
Respect to their own health	70.91	89.67	0.32	5.25	64.44	89.71	0.65	6.38	*0.04*
Trustworthiness/honesty/probity	73.33	93.37	0.31	5.12	76.36	94.24	0.54	5.24	*0.88*
Management/leadership skills	55.56	83.73	0.43	6.98	64.71	84.35	0.76	7.45	*0.62*
Overall ability	64.62	89.68	0.43	6.95	71.67	90.60	0.74	7.23	*0.92*
*Averages*	*62.20*	*88.58*	*0.40*	*6.54*	*67.59*	*89.08*	*0.71*	*6.93*	*0.50*
*Overall for all doctors*	*64.90*	*88.83*	*0.56*	*6.73*					

Note: The maximum scores are 100 except: for PEP GPiT—Awareness of limitations 98.57, and Management/leadership skills 98.33; for AGPT GPiT—Ability to say no 94.00, Awareness of limitations 98.67, Ability to manage stress 98.18, and Management/leadership skills 98.18. Difference calculated as AGPT – PEP. Statistically significant item differences (≤ 0.05) are shown in bold

Colleagues who were doctors gave significantly lower scores (85.93%) than non-doctor colleagues (90.74%, p ≤ 0.001). Female colleagues gave significantly higher scores (89.91%) than male colleagues (86.72%, p ≤ 0.001). Controlling for the effects of colleague and gender showed that 4% of the variance in average score provided by all colleague raters was due to colleague type (adjusted *R*^*2*^ = 0.04) and less than 1% to colleague gender (adjusted *R*^*2*^ = 0.041 in total). Repeating the analysis for PEP and AGPT GPiT separately showed that, for PEP, colleague type contributed 5% of the variance in a colleague score (adjusted *R*^*2*^ = 0.05) and only 1.5% (adjusted *R*^*2*^ = 0.015) for AGPT. For both PEP and AGPT, gender of colleague contributed 0.1%. In all cases the 19 items contributed to the other 95% of variance in scores provided by colleagues.

**Fig. 2:**
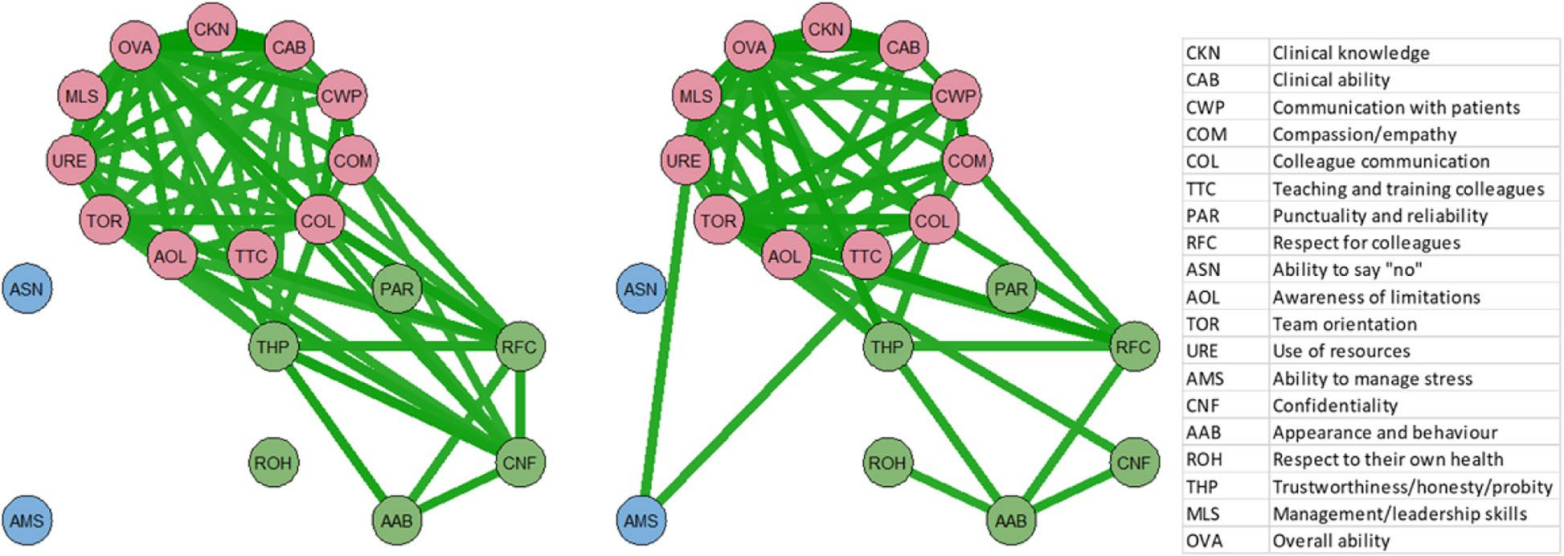
Network visualisation of item interactions based on colleague scores (using Pearson correlations ≥ 0.75) for PEP (left) and AGPT GPiT (right) grouped by PCA component (pink = clinical performance, green = behaviour, blue = self-management)

**Fig. 3:**
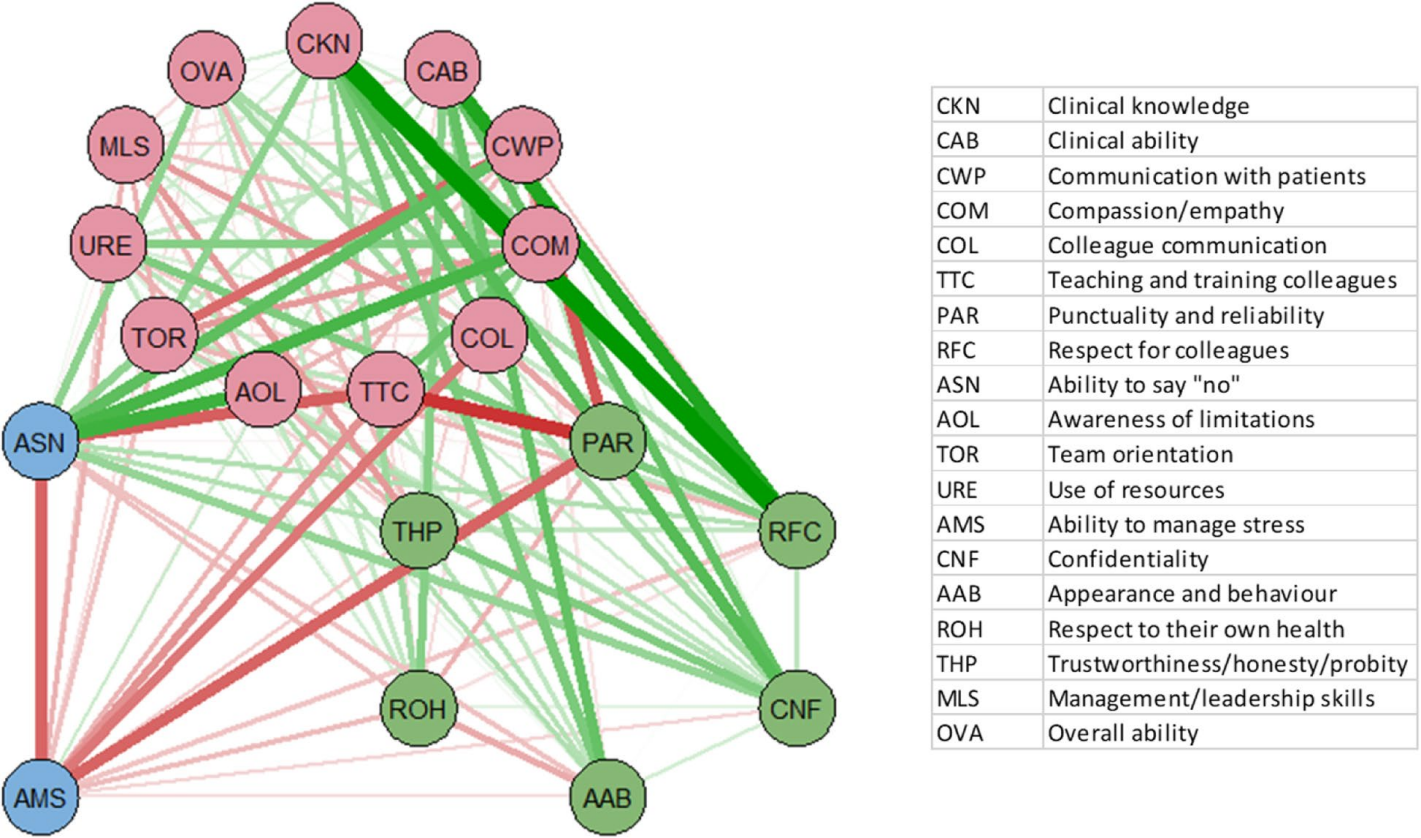
Differences in item interactions (grouped by PCA component) between PEP and APGT doctors (PEP minus AGPT), with green links signifying positive differences for PEP, and red links positive differences for AGPT. Thickness of line signifies strength of difference

Psychometric network analysis was undertaken using inter-item correlations for PEP and AGPT GPiT separately (Fig. 2), revealing strong interactions between clinical performance items (component 1). Finally, to identify possible interventions strategies for training improvement, the inter-item correlations for AGPT GPiT were subtracted from those for PEP GPiT (Fig. 3).

**Fig. 4:**
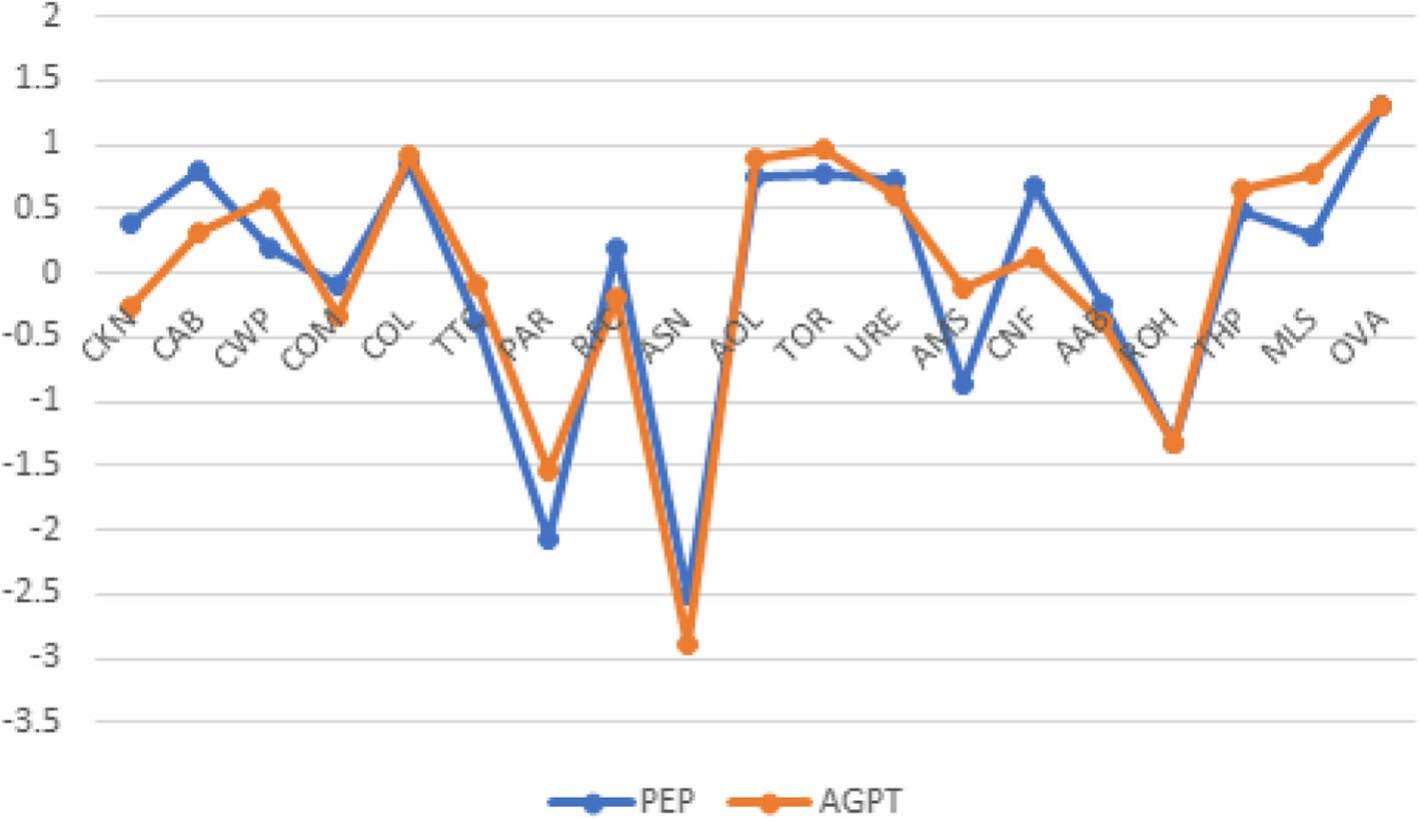
Network node strength for PEP and AGPT networks (Fig. 2) calculated as the standardised values of all summed absolute correlations for each of the 19 items, with z score values on the y-axis. See Fig. 3 for the meaning of the nodes

Node strength (calculated as the z-score standardised sum of absolute inter-item correlations per item) analysis showed that the pattern of connection was broadly similar for both PEP and AGPT GPiT (Fig. 4), providing a measure of network stability. ‘Colleague communication’, ‘Awareness of limitations’, ‘Team orientation’ and ‘Overall ability’ had the strongest connections with other items, whereas ‘Ability to say “no”’ and ‘Punctuality and reliability’ had the weakest.

## Discussion

The results reported here provide an understanding of PEP GPiT MSF performance, including benchmarks, which is an important addition to the literature. In line with the primary aim of this research, the current study is the first systematic examination of the PEP and AGPT GPiT’s performance as rated by colleagues, which is important given that the PEP is a relatively new education program aimed primarily at doctors who gained their medical qualifications outside Australia and were already working in general practice prior to commencing the PEP. The research findings demonstrate that GPiT, regardless of pathway, have very good to excellent skills in the non-clinical domains of practice. While this is the case, the comparative analysis showed there were differences in colleague feedback between PEP and AGPT GPiT groups, in terms of item scores and psychometric networks, that can be used to draw inferences about beneficial additional supports or interventions, as per the secondary aim.

Colleagues tended to give lower scores to PEP GPiT than AGPT GPiT, although the average score received by PEP GPiT (88.58%) was not significantly different from AGPT GPiT (89.08%). When broken down by item, PEP GPiT received 2.13% lower and 1.78% higher scores on two items: Communication with patients and Ability to say ‘no’, respectively (Table 2). The finding regarding colleague scoring of communication with patients supports previous findings of Laurence et al. [13], Bates et al. [12] and Kalra et al. [15]. With respect to ability to say ‘no’, PEP GPiT scored higher than AGPT indicating they are more aware of the need to shape appropriate demand by patients and colleagues and this might reflect the maturity and greater time since obtaining their medical degree that is common for internationally trained doctors [13]. Similarly, it could be associated with the doctor-centric style more common in non-Western countries [15]. It is important to note that saying ‘no’ to patients can be clinically warranted while also leading to lower patient satisfaction, particularly when the patient is denied a referral, pain medication, other new medication, or testing [20].

While female colleagues gave significantly higher scores than male colleagues, controlled regression showed that gender contributed only about 0.1% of the variance in scores provided, with doctor colleagues, both male and female, contributing 5% to scores for PEP GPiT and 1.5% for AGPT GPiT. In other words, doctor colleagues, irrespective of their gender, scored PEP lower than AGPT in this study. These colleague sociodemographic contributions are small in comparison to the 95% contributed by the 19 questionnaire items.

The extraction of the three components of behaviour, clinical performance and self-management is broadly in line with previously peer-established performance categories for experienced GPs receiving colleague feedback for CPD purposes [2, 21]. In particular, clinical performance is strongly associated with patient communication. For experienced GPs, ‘Compassion/empathy’ and ‘Communication with colleagues’ were associated with the behaviour component, whereas for GPiTs in this study, these qualities are associated with clinical performance, according to colleagues. Colleagues perceived that an important aspect of clinical performance of GPiT was good inter-colleague communication as well as ability to show compassion.

The networks (Fig. 2, left and right) show stronger links between behavioural items for PEP GPiTs than for AGPT GPiT, probably due to lengthier medical experience, while AGPT GPiTs show strong links between ‘Ability to manage stress’, ‘Colleague communication’ and ‘Use of resources’. ‘Respect to their own health’ is also more strongly linked to ‘Appearance and behaviour’ for AGPT GPiT. When AGPT interaction values were subtracted from PEP values, AGPT GPiT had stronger interactions between ‘Ability to manage stress’ and ‘Ability to say no’ on the one hand, and ‘Colleague communication’ and ‘Punctuality and reliability’ on the other (Fig. 3). PEP GPiT had stronger links between ‘Compassion/empathy’ and ‘Clinical knowledge’ and ‘Respect for colleagues’. AGPT GPiT had stronger associations between ‘Ability to manage stress’, ‘Use of resources, and ‘Colleague communication’, as well as between ‘Respect to their own health’ and ‘Appearance and behaviour’. Analysis of node strength (Fig. 4) shows that ‘Overall ability’ is highest for both groups, followed by ‘Colleague communication’. There was node strength consistency across most of the items, with PEP GPiT having greater strength in ‘Clinical knowledge’ and ‘Clinical ability’ as well as ‘Confidentiality’. AGPT GPiT had greater strength in ‘Communication with patients’, ‘Ability to manage stress’ and ‘Management/leadership skills’.

These findings have implications for medical education practice. Overall, the performance of PEP GPiT is similar to that of AGPT GPiT and moving forward PEP GPiT can understand their colleague feedback scores from MSF both individually and in comparison with others undertaking the same fellowship program. The findings also indicate that there are certain skills that could be developed further within the PEP GPiT cohort. Based on the findings, PEP GPiT would seemingly benefit from communication training involving colleagues, patients, and in teams. These skills are vital to the provision of patient-centred care that considers not only the patient’s illness or disease, but the patient as a person, including individual experiences, needs, and preferences, to develop a collaborative management plan [22]. Patient-centred care is expected in Australia, but this is not always the model in other countries [23], supporting the need for specific training. Indeed, Yates et al. [24] found that OTDs can have difficulty with nuanced communication that is in line with Australian cultural expectations, and suggests communications training, including pragmalinguistic and sociopragmatic aspects. Further, Wright et al. [25] tested an intervention for OTDs that was designed to support their transition to providing healthcare in Australia, addressing culture and communication, as well as clinical skills. MSF was obtained prior to and after the program, Gippsland Inspiring Professional Standards among International Experts (GIPSIE). Significant improvements were found for three items, clinical skills, teaching and training colleagues, and communication with carers and family. Communication-focused training has also been shown to improve confidence [26]. Communication training could be added to the PEP to address those skills where PEP GPiT showed poorer performance than AGPT GPiT. It seems likely that training that includes simulated consultations with feedback from colleagues that is recorded for later review could be beneficial for OTDs within the PEP. If a program like GIPSIE was to include the pragmalinguistic and sociopragmatic aspects, as suggested by Yates et al. [24], it is possible that this would also improve communication with patients. Further, greater emphasis could be placed on helping PEP GPiT relieve stress by talking more with colleagues and making better use of resources, in line with their AGPT counterparts. Areas where AGPT GPiT could benefit include greater respect for and better communication with colleagues, as well as improvement in clinical ability and skills. These colleague aspects are expected to develop as the doctor gains further training and experience but could also be facilitated through feedback as part of supervision.

This research is, to the authors’ knowledge, the first to investigate the performance of doctors, as rated by their colleagues while undertaking the PEP. This is important due to the prior research suggesting that this group might be at greater risk of underperformance, including in practice and on summative assessments/exams, as well as a greater risk of needing remediation [9, 10, 14, 27, 28]. These findings add further nuances to existing research, as well as indicating areas where targeted intervention is likely to be beneficial. Further, the use of psychometric network analysis with MSF data is novel, depicting the relationships and interactions between each item for each cohort of GPiT.

There were limitations to be considered when interpreting the results. For example, although the item differences are statistically significant, there is very little to distinguish PEP and AGPT GPiT’s performance. Each of these groups demonstrate performance rating percentages around the 90% mark, which reflects the mid-point in the ‘very good’ to ‘excellent’ range. Big data tends to statistically accentuate small differences where they occur, meaning this needs to be accounted for, especially if feedback data is to be used for high-stakes assessment (such as for revalidation of their medical licence, as is required in the United Kingdom by the General Medical Council [29] and in Australia by AHPRA to inform registration conditions), rather than as a tool to facilitate educational feedback. It should also be noted that no attempt was made in this study to compare the content and structure of the two programs. Finally, the focus on the colleague feedback portion of MSF limits the conclusions that can be drawn compared to conclusions utilising the entirety of MSF data, though it could be argued that this would be best presented when all related publications are available.

## Conclusion

The aim of the research was to examine the appraisals of PEP and AGPT GPiT as rated by their colleagues. This research contributes to the bodies of literature for the performance of PEP GPiT and by extension, OTDs. PEP and AGPT GPiT have similar overall feedback ratings, in the range of very good to excellent. Differences were found between the PEP GPiT and AGPT GPiT for 15 items (two statistically significant) within the colleague feedback, reflecting a general trend for PEP GPiT doctors to score lower than AGPT GPiT doctors, with one of the significantly different items being communication with patients. Network analysis revealed both groups of doctors had strongly interconnected clinical performance skills. PEP GPiT had stronger connections among behaviour items and AGPT GPiT dealt better with stress through colleague communication and use of resources. Overall, the findings show that PEP GPiT perform well, showing very good to excellent professionalism, although communication skills could be a focus for further development.

## Data Availability

The data that support the findings of this study are available from CFEP Surveys but restrictions apply to the availability of these data, which were used under license for the current study, and so are not publicly available. Data are however available from A/Professor Michael Greco (email: Michael.Greco@cfepsurveys.com.au) upon reasonable request and with permission of CFEP Surveys.
